# Functional MRI reveals evidence of a self-positivity bias in the medial prefrontal cortex during the comprehension of social vignettes

**DOI:** 10.1093/scan/nsz035

**Published:** 2019-05-14

**Authors:** Eric C Fields, Kirsten Weber, Benjamin Stillerman, Nathaniel Delaney-Busch, Gina R Kuperberg

**Affiliations:** 1 Department of Psychiatry and Athinoula A. Martinos Center for Biomedical Imaging, Massachusetts General Hospital, Charlestown, MA 02129, USA; 2 Department of Psychology, Tufts University, Medford, MA 02155, USA; 3 Department of Psychology, Boston College, Chestnut Hill, MA 02467, USA; 4 Department of Psychology, Brandeis University, Waltham, MA 02453, USA; 5 Max Planck Institute for Psycholinguistics, Nijmegen, The Netherlands; 6 Donders Institute for Brain, Cognition and Behaviour, Radboud University Nijmegen, Nijmegen, The Netherlands; 7 Department of Psychology, New York University, New York, NY 10003, USA

**Keywords:** emotion, valence, superiority illusions, better-than-average effect, optimistic bias, mPFC, self, fMRI

## Abstract

A large literature in social neuroscience has associated the medial prefrontal cortex (mPFC) with the processing of self-related information. However, only recently have social neuroscience studies begun to consider the large behavioral literature showing a strong self-positivity bias, and these studies have mostly focused on its correlates during self-related judgments and decision-making. We carried out a functional MRI (fMRI) study to ask whether the mPFC would show effects of the self-positivity bias in a paradigm that probed participants’ self-concept without any requirement of explicit self-judgment. We presented social vignettes that were either self-relevant or non-self-relevant with a neutral, positive or negative outcome described in the second sentence. In previous work using event-related potentials, this paradigm has shown evidence of a self-positivity bias that influences early stages of semantically processing incoming stimuli. In the present fMRI study, we found evidence for this bias within the mPFC: an interaction between self-relevance and valence, with only positive scenarios showing a self *vs* other effect within the mPFC. We suggest that the mPFC may play a role in maintaining a positively biased self-concept and discuss the implications of these findings for the social neuroscience of the self and the role of the mPFC.

## Introduction

The relationship between emotion and the self-concept lies at the core of human well-being. Understanding this complex relationship is critical for understanding motivation, learning and decision-making (Taylor and Brown, 1988; Dunning *et al.,* 2004; Sharot and Garrett, 2016) in both healthy individuals and in neuropsychiatric disorders (Beck *et al.,* 1979; Frith, 1992; Shestyuk and Deldin, 2010; Holt *et al.,* 2011). It is therefore important that we study the cognitive and neural mechanisms by which the self-concept and self-esteem are constructed and maintained. Here we report a functional MRI (fMRI) study examining the interaction between emotional valence and self-relevance in processing within a region that is classically associated with the self: the medial prefrontal cortex (mPFC).

### The self-positivity bias

It is well established that people tend to view themselves in an unrealistically positive light when compared to others or objective standards. We see ourselves as having more positive (and fewer negative) traits and abilities than others, and we expect more positive outcomes for ourselves across many domains (Taylor and Brown, 1988; Armor and Taylor, 2002; Dunning *et al.,* 2004; Alicke and Govorun, 2005). We are able to maintain these positive self-evaluations via motivated reasoning and asymmetric treatment of positive and negative self-related information. In response to negative information about ourselves, we employ a variety of strategies such as reinterpreting outcomes, shifting standards of comparison, and attributing negative outcomes to external, situation-specific factors (Armor and Taylor, 2002; Mezulis *et al.,* 2004). The result is that beliefs are more likely to be updated in response to positive than negative information about ourselves (Sharot and Garrett, 2016).

This ‘self-positivity bias’ has important real-world consequences. Positive self-views are often seen as key for self-esteem and motivation (Taylor and Brown, 1988; Sharot and Garrett, 2016), and lack of a self-positivity bias is associated with mood disorders (Beck *et al.,* 1979; Shestyuk and Deldin, 2010; Goldin *et al.,* 2013; Garrett *et al.,* 2014). In addition, modeling work suggests that, under many circumstances, unrealistically positive views about the self can lead to adaptive behavior (Johnson and Fowler, 2011). On the other hand, there can be negative consequences of such positive illusions. These include a failure to adjust behavior in response to knowledge of disease risk factors and inadequate studying by students who have an unrealistic perception of their own knowledge (Dunning *et al.,* 2004; Johnson and Fowler, 2011). It is therefore important to understand the mechanisms underlying unrealistic self-positivity effects (Flagan and Beer, 2013; Chavez and Heatherton, 2015).

### Approaches to examining the self-positivity bias in the brain

One way in which researchers have explored the neural basis of the self-positivity bias is to examine brain activity as participants carry out the types of decision-making tasks that are typically used to show self-positivity effects. For example, in a commonly used task, participants explicitly compare themselves to an average peer on various traits. The key finding is that well over half the participants rate themselves above average on positive traits or below average on negative traits, which is of course statistically impossible (Alicke and Govorun, 2005). In a series of fMRI studies, Beer and colleagues reported that the degree to which participants showed the self-positivity bias (e.g. rated themselves above average or claimed knowledge they did not have) was associated with activity within the orbitofrontal cortex (OFC; Beer and Hughes, 2010; Beer *et al.,* 2010; Hughes and Beer, 2012). However, the pattern of activation within this region, as well as its functional connectivity, differed depending on whether self-esteem is under threat (Flagan and Beer, 2013; Hughes and Beer, 2013). Beer and colleagues took this as evidence that behavioral self-positivity effects do not all reflect the same cognitive mechanisms; they can emerge either from simple heuristics and cognitive biases or motivated cognition, depending on the context (Beer, 2014; Beer and Flagan, 2015).

Understanding the neural underpinnings of decision-making processes associated with the self-positivity bias is important because it reveals the mechanisms underlying active self-enhancement. On the other hand, some theorists have argued that self-positivity effects in these kinds of tasks reflect more general cognitive biases and/or the desire to present oneself well, rather than reflecting the participant’s true self-concept (Paulhus, 1993; Farnham *et al.,* 1999; Chambers and Windschitl, 2004; Buhrmester *et al*., 2011; see discussion in Fields and Kuperberg, 2015). These previous studies therefore leave open the question of whether the self-positivity bias emerges purely through processes of explicit self-related decisions, or whether it is also a basic, implicit aspect of the way we view ourselves. If the latter is the case, then the bias should also influence brain regions that are classically associated with self-processing.

A large neuroimaging literature has identified a network associated with processing self-related information. Rather than employing the kinds of social comparison decision-making tasks used to study the self-positivity bias, these studies have more directly examined contrasts between self *vs* other. These include comparisons between conditions in which participants think about themselves *vs* conditions in which they think about others, or in which they are presented with self-relevant vs. other-relevant stimuli. Such contrasts reveal activity within temporal poles, the temporal-parietal junction and much of the cortical midline (Northoff *et al.,* 2006; Legrand and Ruby, 2009; Qin *et al.,* 2013). Within this network, the region most consistently associated with self-related processing is the mPFC, usually in areas dorsal to the orbitofrontal region observed in the social comparison and judgment tasks described above (Northoff *et al.,* 2006; Denny *et al.,* 2012; Wagner *et al.,* 2012; Araujo *et al.,* 2013).^1^ While there is debate about the precise function of this region and the extent to which it is specialized or selective for self-related processing (Northoff and Bermpohl, 2004; Gillihan and Farah, 2005; Uddin *et al.,* 2007; Legrand and Ruby, 2009; Denny *et al.,* 2012; Flagan and Beer, 2013; Qin *et al.,* 2013), its consistent activation by self-related stimuli and conditions suggests that it plays an important role in processing information about the self. It is therefore natural to ask whether self-related activity within the mPFC can be modulated by the self-positivity bias.

Only a handful of fMRI studies have manipulated both valence and self-relevance within the same paradigm, and most of these studies have manipulated self-relevance through the task, for example, by asking participants to judge whether positive or negative trait adjectives or other stimuli are self-relevant *vs* judging whether they are relevant to someone else (Fossati *et al.,* 2003; Fossati *et al.,* 2004; Moran *et al.,* 2006; Phan *et al.,* 2004; see also Ochsner *et al.,* 2004; Lee and Siegle, 2012). Because participants are more likely to judge positive stimuli as self-relevant, this confounds self-relevance with valence.

An alternative approach is to manipulate both the self-relevance and valence of the stimuli themselves to examine how the brain is modulated by the interaction between these two variables during the processing of these stimuli. This type of paradigm can therefore test whether the self-positivity bias is a relatively automatic aspect of how we process information about ourselves.

In a previous fMRI study, Herbert *et al.* (2011b) took this general approach. Participants read short positive and negative phrases that were presented either in the third person or in the first person, e.g. ‘his fear’ *vs* ‘my fear’. First person context increased the effects of valence in emotion-associated regions (e.g. amygdala). The authors also reported differences between first person and third person trials in the mPFC, but this effect did not differ according to valence. However, in contrast with our own previous work (discussed below), a previous study using event-related potentials (ERPs) with the same materials also showed no effects of the self-positivity bias (Herbert *et al.,* 2011a). One reason for this may be the limited context of the two-word noun phrases used. Perhaps more importantly, it is not clear whether phrases in first person should be regarded as truly self-relevant given that participants have a lot of experience hearing and reading sentences in first person (e.g. in conversation, on social media, in novels) without interpreting them as being about themselves. Indeed, previous behavioral work has shown that second person (‘you’) is more likely than first person (‘I’) to lead people to read text as self-relevant (Brunyé *et al.,* 2009; Brunyé *et al.,* 2013; see also Brunyé *et al.,* 2011; Brunyé *et al.,* 2016).

### The present study

We have previously developed a paradigm to probe effects of the self-positivity bias on the processing of self-relevant information in the absence of self-related judgments or decisions (Fields and Kuperberg, 2015). Participants are simply asked to read and comprehend short two-sentence vignettes. Valence is varied by whether the second sentence has a neutral, positive or negative outcome (determined by a single word). Self-relevance is varied by changing the subject of the second sentence from a person’s name to ‘you’, which, as noted above, is known to lead readers to adopt a self-relevant perspective (Brunyé *et al.,* 2009; Brunyé *et al.,* 2011; Brunyé *et al.,* 2013). For example: ‘A man knocks on *Sandra’s*/*your* hotel room door. *Sandra*/*You* see(s) that he has a tray/gift/gun in his hand’. This design therefore fully crosses Valence (neutral, pleasant, unpleasant) and Self-Relevance (self, other). Because this approach gives participants no indication that their self-views are being assessed, it provides a method to examine effects of the self-positivity bias in the absence of explicit self-assessment, and avoids the confounds inherent in manipulating self-relevance via a judgment task.

In a previous ERP study using this paradigm (Fields and Kuperberg, 2015), we examined the N400 component of the ERP, which is reduced to the extent that the semantic features of a word match predictions generated by the preceding context (Kutas and Federmeier, 2011). We showed that positive words elicited a smaller N400 in self-relevant (vs. other-relevant) contexts, while no effects of self-relevance were observed in neutral or negative scenarios. This shows that participants had stronger expectations for positive information in self-relevant scenarios, and that these expectations influenced the earliest stages of semantically processing an incoming word during comprehension. This study therefore provided evidence that the self-positivity bias is a relatively automatic aspect of the way we comprehend self-relevant information.^2^

In the present fMRI study, we used this paradigm to test the hypothesis that activity in the mPFC—a region that, as discussed above, has been strongly associated with self-related processing—would also show effects of the self-positivity bias. We predicted this bias would manifest as a larger effect of self-relevance for the positive scenarios than the negative or neutral scenarios; i.e. the scenarios most consistent with the positively biased self-concept would show the greatest mPFC activation.

## Methods

### Participants

Seventeen female participants were recruited through an advertisement on a Tufts University community website (tuftslife.com). Only female participants were included in order to increase power by reducing heterogeneity and increasing the effect size (exploratory analyses of our ERP data in the same paradigm and population suggested female participants showed larger main effects of the emotion manipulation). Self-reported race and ethnicity was non-Hispanic White (12), Hispanic (1), Asian (1), mixed Asian/White (2) and unreported (1). All participants were right-handed native English speakers between the ages of 18 and 23 (M = 20.7, SD = 1.3), who reported no history of psychiatric or neurological disorders. Participants were paid for their participation and provided informed consent in accordance with the procedures of the Institutional Review Board of Massachusetts General Hospital.

**Table 1:** *Examples of two-sentence scenarios in each of the six conditions.* The critical word is underlined (but did not appear underlined in the actual stimulus lists). Thirty-six scenarios were followed by comprehension questions. For example, the scenario ‘Casper is/You are new on campus. His/Your classmates think he is/you are quite idiosyncratic/clever/dumb compared to others.’ was followed by the question ‘Did Casper/you go to this school last year?’ with the correct answer being ‘no’. Participants were instructed to press a button corresponding to the index finger and middle finger for yes and no respectively before the question left the screen

**Other**			**Self**		
**Positive**	**Neutral**	**Negative**	**Positive**	**Neutral**	**Negative**
A man knocks on Sandra’s hotel room door. She sees that he has a gift in his hand.	A man knocks on Sandra’s hotel room door. She sees that he has a tray in his hand.	A man knocks on Sandra’s hotel room door. She sees that he has a gun in his hand.	A man knocks on your hotel room door. You see that he has a gift in his hand.	A man knocks on your hotel room door. You see that he has a tray in his hand.	A man knocks on your hotel room door. You see that he has a gun in his hand.
Fletcher writes a poem for a class. His friends think it’s a very beautiful composition.	Fletcher writes a poem for a class. His friends think it’s a very intricate composition.	Fletcher writes a poem for a class. His friends think it’s a very boring composition.	You write a poem for a class. Your friends think it’s a very beautiful composition.	You write a poem for a class. Your friends think it’s a very intricate composition.	You write a poem for a class. Your friends think it’s a very boring composition.
Vince spends time with relatives over the break. This turns out to be a wonderful experience for him.	Vince spends time with relatives over the break. This turns out to be a characteristic experience for him.	Vince spends time with relatives over the break. This turns out to be a disastrous experience for him.	You spend time with relatives over the break. This turns out to be a wonderful experience for you.	You spend time with relatives over the break. This turns out to be a characteristic experience for you.	You spend time with relatives over the break. This turns out to be a disastrous experience for you.
After dinner, Lydia is involved in a discussion. She makes a few remarks that impress her friends.	After dinner, Lydia is involved in a discussion. She makes a few remarks that surprise her friends.	After dinner, Lydia is involved in a discussion. She makes a few remarks that hurt her friends.	After dinner, you are involved in a discussion. You make a few remarks that impress your friends.	After dinner, you are involved in a discussion. You make a few remarks that surprise your friends.	After dinner, you are involved in a discussion. You make a few remarks that hurt your friends.
Carmelo has been in his current job for over a year. He learns he is getting a bonus this December.	Carmelo has been in his current job for over a year. He learns he is getting a transfer this December.	Carmelo has been in his current job for over a year. He learns he is getting a pay-cut this December.	You have been in your current job for over a year. You learn you are getting a bonus this December.	You have been in your current job for over a year. You learn you are getting a transfer this December.	You have been in your current job for over a year. You learn you are getting a pay-cut this December.

**Table 2:** Valence and arousal ratings of scenarios. Scenarios were rated by online participants who did not participate in the MRI study. Valence was rated on a scale of 1 (most negative) to 7 (most positive) with 4 as neutral. Arousal was rated on a scale of 1 (least arousing) to 7 (most arousing). Means are presented with standard deviations (across scenarios) in parentheses

	**Other**			**Self**		
	**POS**	**NEU**	**NEG**	**POS**	**NEU**	**NEG**
**Valence**	5.41 (0.51)	4.30 (0.65)	2.30 (0.61)	5.55 (0.60)	4.35 (0.70)	2.26 (0.62)
**Arousal**	3.76 (0.77)	3.34 (0.79)	3.89 (0.83)	4.05 (0.83)	3.57 (0.85)	4.04 (0.85)

### Stimuli

Stimuli were a modified version of those used in our previous ERP work (Fields and Kuperberg, 2012, 2015, 2016). Two hundred sixteen sets of two-sentence scenarios were developed, each with three Valence conditions (positive, neutral, and negative) and two Self-Relevance conditions (self and other) so that there were six versions for each scenario: self-positive, self-neutral, self-negative, other-positive, other-neutral and other-negative.

Example scenarios are presented in Table 1. All scenarios were written in the present tense. The first sentence (4–13 words long) always introduced a situation involving one or more people, only one of which was specifically named (the protagonist, 50% female), and it was always neutral in valence. To create the self conditions, the named person was changed to ‘you’ (Brunyé *et al.,* 2009; Brunyé *et al.,* 2011; Brunyé *et al.,* 2013). The second sentence (8–10 words) continued the scenario and was the same across all Valence conditions except for one word, which was positive, neutral or negative. This critical word was always the either the sixth word (48 scenarios) or the seventh word (168 scenarios) of the second sentence.

#### Valence and arousal ratings

We obtained valence and arousal ratings of all six conditions of the full two-sentence scenarios from online raters (mean = 12.9, range = 8–21 raters per scenario) from Amazon Mechanical Turk. Mean ratings are presented in Table 2.

### Procedure

#### Stimulus presentation and task

Scenarios were divided into six lists with the six conditions counterbalanced across the lists. Each list included 216 sentence pairs (36 in each condition), which were broken into six blocks. Participants were randomly assigned to one of the lists. Stimuli were presented on a projector in white font centered on a black background. Each trial began with a fixation cross of variable duration (most commonly 2 s but ranging up to 20 s) to introduce jitter. Fixation timings were determined using Optseq (https://surfer.nmr.mgh.harvard.edu/optseq). Each sentence of the scenario was presented on the screen for 4 s.

Six comprehension questions were randomly interspersed in each block and appeared for 4 s directly after the second sentence of the scenario. The purpose of these questions was simply to ensure that participants were paying attention and comprehending the scenarios (see Table 1).

#### MRI Acquisition

Structural and fMRI was acquired with a 3T Siemens Trio scanner and 32-channel head coil. FMRI data were acquired over six 7 min and 38 s runs. In each run, 230 functional volumes [36 axial slices (anterior commissure-posterior commissure aligned), 3.2 mm slice thickness, 0.64 mm skip, 200 mm field of view, in-plane resolution of 3.125 mm] were acquired with a gradient echo sequence (TR = 2 s, TE = 25 ms, flip angle = 77°, ascending acquisition order). In addition, at the beginning and end of the scanning session, we acquired two T1-weighted high-resolution structural images (1 mm isotropic multi-echo Magnetization Prepared Rapid Gradient Echo: TR = 2.53 s, flip angle = 7°, four echoes with TE = 1.64 ms, 3.5 ms, 5.36 ms, 7.22 ms). We used the higher quality of the two structural scans from each subject (based on visual inspection) for the subsequent analysis.

#### MRI processing and analysis

Preprocessing, first level and second level analyses of the fMRI data were conducted in SPM8.

The first four images in each run were discarded to eliminate transient non-saturation effects. The next step was to detect spikes and interpolate these bad slices from surrounding images using the ArtRepair toolbox (cibsr.stanford.edu/tools/human-brain-project/artrepair-software.html; Mazaika *et al.,* 2009). On average 0.3% of slices (range 0 to 4.0%) were interpolated. Images were then slice-time corrected and the volumes were realigned to the first image of each run and then to each other. The functional images were aligned with the structural image by co-registering the mean functional image to the structural image. The anatomical images were segmented into gray and white matter, and the spatial normalization parameters acquired during this step were used to normalize the functional images to the International Consortium for Brain Mapping template for European brains. Finally, the images were smoothed with an 8 mm full width at half maximum Gaussian kernel.

We modeled the data using a general linear model with the following regressors: one for fixation, one for the first sentence of each scenario, six for the second sentence of each scenario (one for each condition: Self-Positive, Self-Neutral, Self-Negative, Other-Positive, Other-Neutral and Other-Negative) and one for the comprehension questions. All regressors were convolved with a canonical hemodynamic response function. The realignment parameters for movement correction were also included in the model.

To test our a priori hypotheses concerning the mPFC, we defined a region of interest (ROI) using the anatomical definition of the mPFC in MNI space (|x| < 25, y > 15, z > −5) from Denny *et al.*’s (2012) meta-analysis of self-activations in the mPFC. This ROI includes the ventromedial PFC and dorsomedial PFC, but not the OFC (see footnote 1). To test the interaction of Valence and Self-Relevance within this region, we used a within-subjects ANOVA design matrix that consisted of one regressor for each individual subject and one regressor for the Self vs. Other contrast at each level of Valence. We set an initial voxel-level threshold of *P* < 0.001, and we inferred significance if the peak of any voxel within the region reached a familywise error (FWE)-corrected threshold of *P* < 0.05 using a small volume correction (Worsley *et al.,* 1996). We report the coordinate, z-score and *P*-value of this peak. All reported coordinates are in Montreal Neurological Institute (MNI) space.

Given the work of Beer and colleagues showing an important role for the medial OFC for self-positivity in social comparison tasks (Beer, 2014; Beer and Flagan, 2015), we also conducted an exploratory analysis (using the same model as described above) of a second ROI, the medial OFC, defined as |x| < 25, y > 15, z < −5 in MNI space (i.e. all portions of the medial PFC ventral to the ROI defined above).

In addition to this ROI analysis approach, we carried out whole brain analyses, which are reported in the supplementary materials.

## Results

### Behavioral data

Participants failed to provide a response for 3.1% of comprehension questions (an average of 1.1 of the 36 questions). For the remaining trials, accuracy ranged from 81% to 100% with an average of 91%.

### FMRI results

**Fig. 1:**
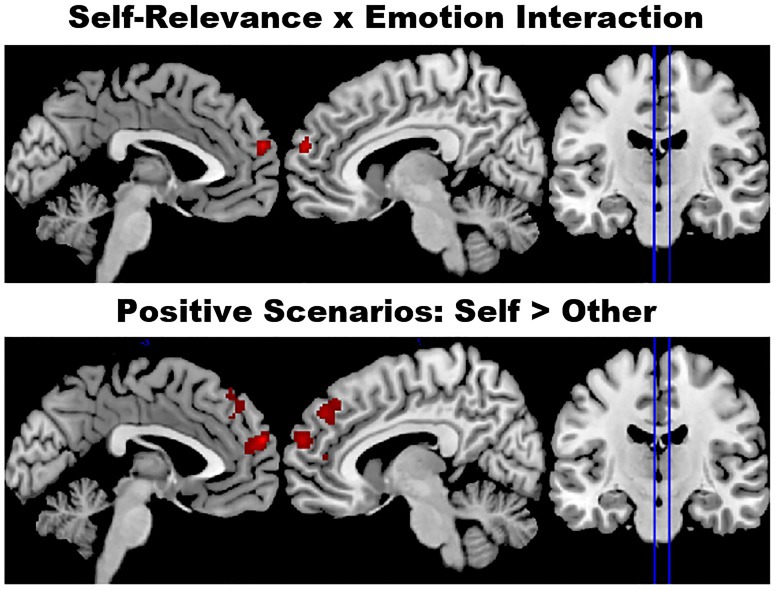
Activations in the mPFC ROI. A Self-Relevance x Emotion interaction was observed in the mPFC small volume correction analysis. Follow-ups showed effects of Self-Relevance for positive scenarios, but not neutral or negative scenarios. Voxels showing greater activity for self than other are highlighted in red (no regions showed the opposite effect). Effects are shown at a voxel-level significance threshold of *P* < 0.001 for regions where the peak reached a FWE-corrected threshold of *P* < 0.05. See Table 2 for the full list of peaks.

The small volume analysis in the mPFC ROI revealed a significant Valence x Self-Relevance interaction (peak MNI coordinates [0, 60, 22]; peak voxel level *p*_FWE_ = 0.047 small volume corrected, z-score = 4.26), see Figure 1. There were no significant main effects of Valence or Self-Relevance within this region.

We followed up the interaction by examining all pairwise contrasts with small volume correction in the mPFC ROI. In line with our predictions, follow-ups showed that self-relevant material elicited greater activation than other-relevant material for positive scenarios, but not for neutral or negative scenarios. This self-other effect within positive scenarios emerged in a cluster closely overlapping with the cluster that showed the interaction effect; the respective peaks were observed at [−2, 60, 22] and [0, 60, 22]) and 98% of the voxels in the interaction cluster were significant in the pairwise contrast. Effects were also seen in more dorsal areas of mPFC (see Figure 1 and Table 3).

**Table 3:** Self-positive *vs* other-positive activations in the mPFC ROI

**R/L**	**Peak voxel** ***P*-value**	**z-score**	**MNI (x, y, z)**	**Cluster level**
L	0.003	5.10	−2, 60, 22	*P*(FWE) < 0.001,k = 506
L	0.009	4.80	−6, 62, 24	
R	0.066	4.24	8, 38, 46	*P*(FWE) = 0.001,k = 235
R	0.090	4.14	6, 46, 40	

We also examined pairwise valence contrasts within the self-relevant and other-relevant conditions. Here, the only significant activation was a cluster showing greater activity for negative than positive scenarios within the other condition (peak MNI coordinates [14, 60, 24], peak voxel level *p*_FWE_ = 0.002 small volume corrected, z-score = 5.14). Notably, this effect showed only partial overlap with the interaction effect; the peak was not included in the interaction cluster and only 53% of the voxels in the interaction cluster were significant in the pairwise contrast (with 69% of significant voxels from the pairwise contrast falling outside the interaction cluster).^3^

Within the medial OFC ROI, no significant main effects or interactions emerged, all *p*s < 0.19.

Whole brain analyses comparing each condition to baseline as well as the full ANOVA design are reported in the supplementary materials.

## Discussion

In the present study, we showed that the mPFC—a region that has long been associated with the processing or representation of the self (Northoff *et al.,* 2006; Denny *et al.,* 2012; Wagner *et al.,* 2012; Araujo *et al.,* 2013)—is sensitive to the self-positivity bias. Specifically, when participants read self-relevant and other-relevant social vignettes, without any requirement to make an explicit decision about self-relevance, we found an interaction between self-relevance and valence, with only the positive scenarios showing more activity to self-relevant than other-relevant scenarios.

### A self-positivity bias in the mPFC

This effect of the self-positivity bias was observed in the area of the mPFC that has been most strongly associated with self-related cognition (cf. Denny *et al.,* 2012). Although there is disagreement about the precise function of the mPFC and the degree to which it is specialized or specific for self-related (or social) processing (Northoff and Bermpohl, 2004; Uddin *et al.,* 2007; Legrand and Ruby, 2009; Saxe, 2009; Zaki and Ochsner, 2011; Denny *et al.,* 2012), it is consistently modulated by self-related experimental manipulations (Northoff *et al.,* 2006; Legrand and Ruby, 2009; Denny *et al.,* 2012; Araujo *et al.,* 2013; Qin *et al.,* 2013). We therefore interpret our findings as supporting the idea that a core aspect of self-related processing is engaged to a greater degree when information matches positive self-views. This adds to evidence of representational similarity between positive valence and the self within ventral mPFC (Chavez *et al.,* 2017) to suggest that the self-positivity bias is a basic, implicit aspect of the way we view the world.

The present results complement the findings of previous neuroimaging studies that have focused on how the self-positivity bias emerges in explicit social comparison tasks or tasks that require some kind of potentially self-enhancing judgment. These studies have highlighted the role of the OFC in such decision-making processes (reviewed in Beer, 2014; Beer and Flagan, 2015). In the present study, we did not find modulation of the orbitofrontal region. This, however, is not necessarily surprising, given that participants were not making any such decisions or judgments. The findings described here suggest that when participants are simply comprehending information about themselves, without making any judgments about themselves, neural effects of the self-positivity bias can manifest in a more dorsal region that is classically associated with self-processing.

### Functional role of the mPFC in instantiating the self-positivity bias

The pattern of effects observed in the present study is consistent with that seen in our previous ERP study using the same stimuli (Fields and Kuperberg, 2015). In that study, we also observed a significant effect of self-relevance in the positive, but not the neutral or negative scenarios. This effect was seen on the N400 component, suggesting that self-relevant scenarios generated predictions for positive information. We think that it is unlikely that the mPFC modulation observed in this fMRI study and the modulation previously observed on the N400 reflect precisely the same underlying neural activity or mechanisms. The mPFC is not generally thought to be a source of the N400, and due to their differing spatial and temporal sensitivities, ERP and fMRI often reveal different aspects of the neural response (Lau *et al.,* 2013).^4^ Instead, we suggest that the mPFC modulation observed in the present study may reflect downstream processes that relate to the construction and maintenance of the self-positivity bias.

Behavioral work shows that we are more likely to update our beliefs about ourselves in response to positive than negative information (reviewed by Sharot and Garrett, 2016). This is an important way in which unrealistic self-positivity is maintained in the face of a disconfirming reality (Armor and Taylor, 2002). Interestingly, some previous fMRI studies examining how unrealistic optimism is maintained have linked mPFC activity specifically to belief updating in response to positive self-related information. Sharot *et al.* (2011) asked participants to estimate their likelihood of experiencing various adverse events before presenting the actual average probability of each event. After this task, they reassessed participants’ estimates of the likelihood of each event. They replicated findings (e.g. Eil and Rao, 2011) that participants were unrealistically optimistic and that they updated their beliefs less in response to unexpectedly negative information than unexpectedly positive information. In addition, they found that the same part of mPFC that showed the interaction observed in the present study was related to tracking prediction errors and belief updating specifically for unexpectedly positive (but not negative) feedback. Garrett *et al.* (2014) replicated these results and extended them to people with major depressive disorder (see also Sharot and Garrett, 2016 for general discussion).

Further support for the idea that the mPFC may play an important role in constructing and maintaining the self-positivity bias comes from work on the neural basis of self-esteem. Chavez and Heatherton (2015) have shown that functional connectivity between mPFC and ventral striatum is associated with state self-esteem, and structural connectivity between these regions is associated with trait self-esteem, both at the time of scanning and eight months later (Chavez and Heatherton, 2017).

Although these possibilities are intriguing, it is important to note that the present paradigm does not allow for strong conclusions about the precise cognitive mechanisms represented by the mPFC activation we observed. Indeed, the mPFC has been implicated in many other processes. For example, it is also thought to play an important role in self-projection and counterfactual thinking (Buckner and Carroll, 2007; Spreng *et al.,* 2009). Thus, it is possible that the increased activity for the self-positive scenarios arose because participants were most likely to imagine themselves experiencing or acting out these scenarios.

### Limitations and future directions

It is important to mention some limitations of the current work. First, our sample size of 17 participants was relatively small. Although this is somewhat mitigated by the relatively large number of scenarios and ROI analysis approach, the results should be treated as somewhat preliminary until confirmed or extended in a high-powered study (Button *et al.,* 2013). In addition, our sample was all females, mostly white, between the ages of 18 and 23, and all were students at an elite university. This means that we should be cautious about generalizing these findings. Although work on the self-positivity bias has generally not revealed significant gender differences (Alicke and Govorun, 2005), the bias is likely to be particularly sensitive to other social and cultural differences (Heine *et al.,* 1999; Sedikides *et al.,* 2005; Henrich *et al.,* 2010; Kitayama and Park, 2014). As we have noted previously (Fields and Kuperberg, 2015), we believe the paradigm presented her may be valuable for future research investigating such differences.

## Conclusion

In conclusion, our findings suggest that the mPFC, a region that has long been associated with the representation and processing of self-related information, is modulated by the self-positivity bias in a paradigm that probes self-relevant comprehension, but that does not require explicit decision-making or judgments about the self. Future research should continue to explore the neural mechanisms underlying the self-positivity bias and explore the implications for a social neuroscientific understanding of the self (see also Beer, 2014; Beer and Flagan, 2015; Chavez and Heatherton, 2015; Chavez *et al.,* 2017).

## Supplementary Material

scan-18-185-File006_nsz035

## Footnotes

1 The medial PFC is often divided into the dorsomedial prefrontal cortex (dmPFC), the ventromedial prefrontal cortex (vmPFC), and the orbitofrontal cortex (e.g. Northoff *et al.,* 2006; Beer and Flagan, 2015). These terms broadly correspond to the medial portions of Brodmann area (BA) 9, BA 10 and BA 11, respectively. Self-related effects have been observed in all these regions (Northoff *et al.,* 2006; Denny *et al.,* 2012), but most commonly in vmPFC and dmPFC. Some work suggests that portions of the mPFC in BA 10 around the frontal pole are more strongly associated with self while more dorsal regions are associated with social processing more generally (Denny *et al.,* 2012). However, the region suggested to be most strongly associated with self-processing is near where the border of vmPFC and dmPFC is often placed, with individual studies showing effects on both sides. In the present study, we therefore use the term mPFC to refer to combined vmPFC and dmPFC, while always referring to the orbitofrontal cortex separately by its name.

2 Some readers may question the term ‘bias’ here since there is no objective measure of accuracy for expectations or reactions related to fictional scenarios. However, many classic demonstrations for the self-positivity bias also do not have an objective measure of the behavior in question. For example, Svenson’s (1981) classic finding that over 90% of drivers place themselves in the top 50% in terms of driving skill is taken as a demonstration of the self-positivity bias not because it could be compared to the participants’ actual driving ability, but because it is statistically unlikely that all of the participants who thought they were above average actually were superior drivers. In our paradigm, across a broad range of situations, participants were more likely to expect positive things for themselves when compared to a variety of differently named strangers. Since the third person scenarios were each about a unique individual with no additional background information, we assumed these protagonists (on average) would be interpreted as an ‘average’ peer. It is in this sense that we take the positivity effect shown in this paradigm as a manifestation of the better-than-average effect/optimistic bias.

3 Based on these contrast results, one could argue that the interaction was partially driven by reduced activity to the other-positive condition, rather than, or in addition to, increased activity to the self-positive condition as predicted. It is not clear, however, what would explain such an effect. Although some work has suggested that the self-positivity bias can result from downward comparison (viewing others less positively; Perloff and Fetzer, 1986), other work suggests that we generally view others, especially individuated others, positively and simply view ourselves more positively (Alicke *et al.,* 1995). In any case, it seems unlikely that participants would have any motivation to view others negatively given that there was no comparison or judgment task in the present study. Given the partial overlap with the interaction effect, we hesitate to interpret this *post hoc* finding.

4 An alternative possibility is that the effect we observed in our previous ERP study did not actually reflect N400 activity, and that what appeared to be a reduced negativity, was in fact an increased positivity that overlapped closely with the timing of the N400. Under this interpretation, the mPFC modulation observed here may have reflected the same neural activity as observed in this previous ERP study.
