# Supplementary material for: Functional MRI reveals evidence of a self-positivity bias in the medial prefrontal cortex during the comprehension of social vignettes
Source: Soc Cogn Affect Neurosci. 2019 May 14;14(6):613–21. doi: 10.1093/scan/nsz035 (PMC6688454; doi:10.1093/scan/nsz035)
Supplement: scan-18-185-File006_nsz035 [file scan-18-185-file006_nsz035.docx]

# Whole Brain Analyses

Whole brain analyses were conducted (a) to visualize activation of the sentences against baseline (fixation) and (b) to explore any other regions outside of our ROIs that might show interactions between Valence and Self-Relevance. For these analyses, we set a voxel-level threshold of p < 0.001, and we report any cluster that was significant at a cluster-level FWE-corrected threshold of p < 0.05.

When compared to baseline, all six conditions activated visual areas in the occipital and temporal lobes as well as a large network of regions generally associated with sentence processing including lateral temporal lobe and ventrolateral prefrontal cortex. These results are shown below in Tables S1-S6.

The 3 x 2 ANOVA examining all six conditions did not reveal significant effects of Valence, Self-Relevance, or a Valence x Self-Relevance interaction that reached cluster-level significance in any region in the whole brain analysis.

**Table S1: Other-Neutral versus Fixation (whole brain)**

| **Region** | **R/L** | **Peak voxel p-value** | **z-score** | **MNI (x, y, z)** | **Cluster level p-value (FWE)** |
| --- | --- | --- | --- | --- | --- |
| Supplementary motor area | -- | 0.0006 | 5.82 | 0, 10, 58 | p(FWE) < 0.0001,  k = 565 |
| Inferior frontal gyrus (pars triangularis) | L | 0.0301 | 4.89 | -50, 24, 4 | p(FWE) < 0.0001,  k = 4426 |
| Middle temporal cortex (anterior) | L | 0.0011 | 5.67 | -52, 4, -20 |  |
| Middle temporal cortex (pole) | L | 0.0745 | 4.65 | -40, 18, -32 |  |
| Precentral gyrus | L | 0.0039 | 5.39 | -38, 0, 50 | p(FWE) = 0.0001,  k = 860 |
| Occipital cortex (calcarine) | R | 0.0515 | 4.75 | 18, -66, 10 | p(FWE) < 0.0001,  k = 1890 |

**Table S2: Other-Positive versus Fixation (whole brain)**

| **Region** | **R/L** | **Peak voxel *p*-value** | **z-score** | **MNI (x, y, z)** | **Cluster level *p*-value (FWE)** |
| --- | --- | --- | --- | --- | --- |
| Inferior frontal gyrus (pars triangularis) | L | 0.0540 | 4.80 | -54, 22, 6 | *p*(FWE) < 0.0001,  k = 4370 |
| Middle temporal cortex (anterior) | L | 0.0001 | 6.21 | -54, -4, -16 |  |
| Occipital cortex (lingual) | L | 0.0161 | 5.11 | -14, -30, -4 | *p*(FWE) = 0.0437,  k = 228 |
| Supplementary motor area | L | 0.0165 | 5.11 | -4, 12, 56 | *p*(FWE) = 0.0009,  k = 516 |
| Occipital cortex (lateral) | L | 0.0324 | 4.94 | -16, -94, -8 | *p*(FWE) < 0.0001,  k = 4675 |
| Precentral gyrus | L | 0.0771 | 4.71 | -38, -2, 54 | *p*(FWE) < 0.0001,  k = 969 |
| Middle temporal cortex (pole) | R | 0.0813 | 4.69 | 56, 6, -16 | *p*(FWE) < 0.0001,  k = 773 |

**Table S3: Other-Negative versus Fixation (whole brain)**

| **Region** | **R/L** | **Peak voxel *p*-value** | **z-score** | **MNI (x, y, z)** | **Cluster level *p*-value (FWE)** |
| --- | --- | --- | --- | --- | --- |
| Inferior frontal gyrus (pars opercularis) | L | 0.0113 | 5.09 | -56, 18, 18 | *p*(FWE) < 0.0001,  k = 5760 |
| Inferior frontal gyrus (pars triangularis) | L | 0.0090 | 5.14 | -58, 20, 24 |  |
| Inferior frontal gyrus (pars orbitalis) | L | 0.0038 | 5.34 | -46, 24, -6 |  |
| Superior temporal cortex (pole) | L | 0.0161 | 5.00 | -46, 8, -22 |  |
| Middle temporal cortex (posterior) | L | 0.0639 | 4.64 | -56, -48, 14 |  |
| Middle temporal cortex (anterior) | L | 0.0003 | 5.89 | -54, -26, -4 |  |
| Middle temporal cortex (anterior) | R | 0.0449 | 4.73 | 62, 0, -16 | *p*(FWE) < 0.0001,  k = 1906 |
| Middle temporal cortex (pole) | R | 0.0186 | 4.96 | 44, 18, -30 |  |
| Supplementary motor area | L | 0.0782 | 4.58 | -2, 10, 62 | *p*(FWE) = 0.0003,  k = 921 |
| Superior frontal cortex (medial) | L | 0.0328 | 4.82 | -8, 60, 42 |  |
| Precentral gyrus | L | 0.0358 | 4.79 | -38, -2, 56 | *p*(FWE) = 0.0008,  k = 781 |
| Occipital cortex (calcarine) | R | 0.0568 | 4.67 | 20, -90, -2 | *p*(FWE) < 0.0001,  k = 2113 |
| Occipital cortex (lateral) | L | 0.0676 | 4.62 | -34, -92, -4 | *p*(FWE) < 0.0001,  k = 2481 |

**Table S4: Self-Neutral versus Fixation (whole brain)**

| **Region** | **R/L** | **Peak voxel *p*-value** | **z-score** | **MNI (x, y, z)** | **Cluster level *p*-value (FWE)** |
| --- | --- | --- | --- | --- | --- |
| Inferior frontal gyrus (pars opercularis) | L | 0.0030 | 5.49 | -52, 18, 20 | *p*(FWE) < 0.0001,  k = 4967 |
| Inferior frontal gyrus (pars triangularis) | L | 0.0098 | 5.22 | -54, 22, 2 |  |
| Inferior frontal gyrus (pars orbitalis) | L | 0.0207 | 5.04 | -48, 24, -8 |  |
| Middle temporal cortex (posterior) | L | 0.0459 | 4.83 | -56, -44, 4 |  |
| Middle temporal cortex (anterior) | L | 0.0003 | 5.94 | -54, -4, -16 |  |
| Precentral gyrus | L | 0.0129 | 5.15 | -38, 0, 50 | *p*(FWE) < 0.0001,  k = 1104 |
| Occipital cortex (lingual) | L | 0.0553 | 4.78 | -20, -88, -14 | *p*(FWE) < 0.0001,  k = 2460 |
| Occipital cortex (lateral) | L | 0.0217 | 5.03 | -24, -104, -2 |  |
| Occipital cortex (calcarine) | R | 0.0852 | 4.67 | 20, -90, -2 | *p*(FWE) < 0.0001,  k = 1876 |
| Occipital cortex (lateral) | R | 0.0251 | 4.99 | 40, -86, -8 |  |
| Superior frontal cortex (lateral) | L | 0.0547 | 4.79 | -14, 32, 64 | *p*(FWE) = 0.0028,  k = 444 |

**Table S5: Self-Positive versus Fixation (whole brain)**

| **Region** | **R/L** | **Peak voxel *p*-value** | **z-score** | **MNI (x, y, z)** | **Cluster level *p*-value (FWE)** |
| --- | --- | --- | --- | --- | --- |
| Inferior frontal gyrus (pars triangularis) | L | 0.0228 | 4.98 | -52, 22, 0 | *p*(FWE) < 0.0001,  k = 5426 |
| Inferior frontal gyrus (pars orbitalis) | L | 0.0192 | 5.02 | -50, 24, -4 |  |
| Superior temporal cortex (pole) | L | 0.0728 | 4.67 | -44, 10, -22 |  |
| Middle temporal cortex (posterior) | L | 0.0031 | 5.45 | -54, -30, -2 |  |
| Middle temporal cortex (anterior) | L | 0.0022 | 5.53 | -52, -4, -18 |  |
| Precentral gyrus | L | 0.0026 | 5.49 | -44, -16, 62 | *p*(FWE) < 0.0001,  k = 1442 |
| Supplementary motor area | R | 0.0158 | 5.07 | 2, 10, 60 | *p*(FWE) < 0.0001,  k = 1091 |
| Superior frontal cortex (medial) | L | 0.0331 | 4.88 | -6, 64, 28 |  |
| Occipital cortex (calcarine) | R | 0.0323 | 4.89 | 18, -90, 2 | *p*(FWE) < 0.0001,  k = 5228 |
| Occipital cortex (lingual) | L | 0.0694 | 4.69 | -20, -90, -14 |  |
| Occipital cortex (lateral) | R | 0.0608 | 4.72 | 40, -86, -8 |  |
| Superior temporal cortex (pole) | R | 0.0696 | 4.69 | 42, 12, -24 | *p*(FWE) < 0.0001,  k = 1028 |

**Table S6: Self-Negative versus Fixation (whole brain)**

| **Region** | **R/L** | **Peak voxel *p*-value** | **z-score** | **MNI (x, y, z)** | **Cluster level *p*-value (FWE)** |
| --- | --- | --- | --- | --- | --- |
| Inferior frontal gyrus (pars opercularis) | L | 0.0938 | 4.64 | -50, 18, 20 | *p*(FWE) < 0.0001,  k = 5855 |
| Inferior frontal gyrus (pars orbitalis) | L | 0.0025 | 5.54 | -46, 24, -4 |  |
| Superior temporal cortex (pole) | L | 0.0846 | 4.67 | -46, 24, -14 |  |
| Middle temporal cortex (anterior) | L | 0.0000 | 6.78 | -52, -8, -18 |  |
| Middle temporal cortex (pole) | L | 0.0022 | 5.57 | -44, 12, -30 |  |
| Inferior temporal cortex (anterior) | L | 0.0796 | 4.68 | -44, -8, -30 |  |
| Precentral gyrus | L | 0.0002 | 5.97 | -40, 0, 58 | *p*(FWE) < 0.0001,  k = 929 |
| Superior temporal cortex (pole) | R | 0.0052 | 5.37 | 46, 12, -22 | *p*(FWE) < 0.0001,  k = 1419 |
| Middle temporal cortex (anterior) | R | 0.0007 | 5.79 | 54, 4, -18 |  |
| Middle temporal cortex (pole) | R | 0.0756 | 4.70 | 48, 18, -34 |  |
| Supplementary motor area | L | 0.0223 | 5.02 | -4, 10, 56 | *p*(FWE) < 0.0001,  k = 1419 |
| Superior frontal cortex (medial) | L | 0.0202 | 5.04 | -10, 56, 46 |  |
| Occipital cortex (lingual) | L | 0.0349 | 4.91 | -20, -90, -12 | *p*(FWE) < 0.0001,  k = 2698 |
| Occipital cortex (lateral) | L | 0.0226 | 5.02 | -16, -96, -8 |  |
| Occipital cortex (calcarine) | R | 0.0817 | 4.68 | 20, -88, 0 | *p*(FWE) < 0.0001,  k = 2117 |
| Occipital cortex (lateral) | R | 0.0312 | 4.93 | 42, -86, -8 |  |

**Table S7: Each Condition versus Fixation in the mPFC ROI**

| **Region** | **R/L** | **Peak voxel *p*-value** | **z-score** | **MNI (x, y, z)** | **Cluster level *p*-value (FWE)** |
| --- | --- | --- | --- | --- | --- |
| Self Relevant Positive – Fixation | | | | | |
| Superior frontal cortex (lateral) | L | 0.0266 | 4.34 | -10, 60, 38 | *p*(FWE) < 0.0001, k = 727 |
| Supplementary motor area | L | 0.0487 | 4.16 | -8, 18, 68 |  |
| Superior frontal cortex (medial) | L | 0.0036 | 4.88 | -6, 64, 28 |  |
| Self Relevant Neutral – Fixation | | | | | |
| Superior frontal cortex (lateral) | L | 0.0059 | 4.79 | -14, 32, 64 | *p*(FWE) = 0.0003, k = 444 |
| Superior frontal cortex (medial) | L | 0.0187 | 4.48 | -10, 64, 26 |  |
| Self Relevant Negative – Fixation | | | | | |
| Superior frontal cortex (lateral) | L | 0.0021 | 5.04 | -10, 56, 46 | *p*(FWE) < 0.0001, k = 970 |
| Supplementary motor area | L | 0.0506 | 4.18 | -4, 18, 70 |  |
| Superior frontal cortex (medial) | L | 0.0063 | 4.77 | -8, 60, 26 |  |
| Other Relevant Positive – Fixation | | | | | |
| Supplementary motor area | L | 0.0122 | 4.61 | -8, 18, 66 | *p*(FWE) = 0.0165, k = 157 |
| Superior frontal cortex (lateral) | L | 0.0202 | 4.47 | -12, 58, 42 | *p*(FWE) = 0.0359, k = 115 |
| Other Relevant Neutral – Fixation | | | | | |
| Superior frontal cortex (medial) | L | 0.0523 | 4.11 | -10, 64, 30 | *p*(FWE) = 0.0433, k = 123 |
| Other Relevant Negative – Fixation | | | | | |
| Superior frontal cortex (lateral) | L | 0.0857 | 3.89 | -12, 36, 62 | *p*(FWE) = 0.0006, k = 562 |
| Supplementary motor area | L | 0.0111 | 4.51 | -8, 24, 70 |  |
| Superior frontal cortex (medial) | L | 0.0035 | 4.82 | -8, 60, 42 |  |
